# Identifying a ten-microRNA signature as a superior prognosis biomarker in colon adenocarcinoma

**DOI:** 10.1186/s12935-019-1074-9

**Published:** 2019-12-30

**Authors:** Rong Ma, Yanyun Zhao, Miao He, Hongliang Zhao, Yifan Zhang, Shuqi Zhou, Mengcong Gao, Di Di, Jue Wang, Jian Ding, Minjie Wei

**Affiliations:** 1 Department of Pharmacology, School of Pharmacy, China Medical University, No.77 Puhe Road, Shenyang North New Area, Shenyang, 110122 Liaoning China; 2 Liaoning Engineering Technology Research Center, China Medical University, No. 77 Puhe Road, Shenyang North New Area, Shenyang, 110122 Liaoning People’s Republic of China; 3 Division of Anti-tumor Pharmacology, State Key Laboratory of Drug Research, Shanghai Institute of Materia Medica, Chinese Academy of Sciences, Shanghai, China

**Keywords:** Colon adenocarcinoma, MicroRNAs, Biomarker, Prognosis

## Abstract

**Background:**

Increasing studies have suggested that aberrant expression of microRNAs might play essential roles in the progression of cancers. In this study, we sought to construct a high-specific and superior microRNAs signature to improve the survival prediction of colon adenocarcinoma (COAD) patients.

**Methods:**

The genome-wide miRNAs, mRNA and lncRNA expression profiles and corresponding clinical information of COAD were collected from the TCGA database. Differential expression analysis, Kaplan–Meier curve and time-dependent ROC curve were calculated and performed using R software and GraphPad Prism7. Univariate and multivariate Cox analysis was performed to evaluate the prognostic ability of signature. Functional enrichment analysis was analyzed using STRING database.

**Results:**

We identified ten prognosis-related microRNAs, including seven risky factors (hsa-miR-197, hsa-miR-32, hsa-miR-887, hsa-miR-3199-2, hsa-miR-4999, hsa-miR-561, hsa-miR-210) and three protective factors (hsa-miR-3917, hsa-miR-3189, hsa-miR-6854). The Kaplan–Meier survival analysis showed that the patients with high risk score had shorter overall survival (OS) in test series. And the similar results were observed in both validation and entire series. The time-dependent ROC curve suggested this signature have high accuracy of OS for COAD. The Multivariate Cox regression analysis and stratification analysis suggested that the ten-microRNA signature was an independent factor after being adjusted with other clinical characteristics. In addition, we also found microRNA signature have higher AUC than other signature. Furthermore, we identified some miRNA-target genes that affect lymphatic metastasis and invasion of COAD patients.

**Conclusion:**

In this study, we established a ten-microRNA signature as a potentially reliable and independent biomarker for survival prediction of COAD patients.

## Background

Colon adenocarcinoma (COAD), the fourth most commonly malignant cancer, has been the fifth leading cause of cancer-related death diseases in worldwide. It was estimated that nearly 101,420 new COAD cases were diagnosed and 27,640 deaths in the United States in 2019 [1]. Despite diagnostic methods and comprehensive treatment have been developed during the past few years, the overall 5-year survival rate of COAD patients is still unsatisfactory and the underlying molecular mechanisms of COAD progression are still elusive [2]. Although several biomarkers for COAD have been undergoing or tested in clinical trials, such as carcinoembryofnic antigen (CEA) [3] and so on, many more potential reliable and valuable biomarkers are imperative to be detected and constructed to improve the prognosis of patients with COAD.

MicroRNAs (miRNAs) are a large family of post-transcriptional regulators that are approximately 21 nucleotides in length and control many developmental and cellular processes in eukaryotic organisms [4]. More recently, increasing studies have demonstrated that microRNAs play essential roles in the progression of cancers. Some microRNAs were associated with disease prognosis and clinical outcome, suggesting microRNAs could be one of the best candidates as potential biomarkers for cancers [5, 6].

In addition, compared with a single biomarker, integrating multiple signature model would fundamentally improve the precise of prognostic value [7], and multigene-expression signatures have been reported to predict prognostic in various cancers [8, 9]. Therefore, searching a panel of microRNA signature might have predictive and prognostic value in patients with COAD.

In the present study, we established an effective microRNAs-based signature to predict the prognosis of COAD taking advantage of TCGA database. And we verified the predictive power of this signature for COAD in validation and entire series. More significantly, we verified our microRNA signature perform better than other signature. Finally, we identified some miRNA-target genes and signaling pathways that promote the progression of COAD patients.

## Materials and methods

### COAD patients’ information collection

The microRNAs, mRNA and lncRNA expression profiles of 441 COAD tissues and eight adjacent tissue samples, and their corresponding clinical information were download from The Cancer Genome Atlas of the National Cancer Institute (TCGA, http://cancergenome.nih.gov). The downloaded clinical information summarized in Table 1.

**Table 1:** Clinical characteristics of COAD in each data set

Characteristics	Test series	Validation series	Entire series
Gender			
Male	157/300 (52.3)	74/141 (52.5)	231/441 (52.4)
Female	143/300 (47.7)	67/141 (47.5)	210/441 (47.6)
Age(years)			
≥ 68	166/300 (55.3)	71/141 (50.4)	237/441 (53.7)
< 68	134/300 (44.7)	70/141(49.6)	204/441 (46.3)
TNM stage			
I	55/290 (19.0)	18/140 (12.9)	73/430 (17.0)
II	113/290 (39.0)	55/140 (39.3)	168/430 (39.1)
III	82/290 (28.3)	42/140 (30.0)	124/430 (28.8)
IV	40/290 (13.8)	25/140 (17.9)	65/430 (15.1)
Race			
Non-white	47/191 (24.6)	24/92 (26.1)	71/283 (25.1)
White	144/191 (75.4)	68/92 (73.9)	212/283 (74.9)
T stage			
T1	8/299 (2.7)	3/141 (2.1)	11/440 (2.5)
T2	55/299 (18.4)	20/141 (14.3)	74/440 (16.8)
T3	200/299 (66.9)	100/141 (70.9)	300/440 (68.2)
T4	36/299 (12.0)	18/141 (12.8)	55/440 (12.5)
N stage			
N0	179/300 (59.7)	78/141 (55.3)	257/441 (58.3)
N1	70/300 (23.3)	34/141 (24.1)	104/441 (23.6)
N2	51/300 (17.0)	29/141 (20.6)	80/441 (18.1)
M stage			
M0	219/259 (84.6)	101/126 (80.2)	320/385 (83.1)
M1	40/259 (15.4)	25/126 (19.8)	65/385 (16.9)
Lymphatic invasion			
No	172/270 (63.7)	71/129 (55.0)	243/399 (60.9)
Yes	98/270 (36.3)	58/129 (45.0)	156/399 (39.1)
Microsatallite instability			
No	58/65 (89.2)	22/26 (84.6)	80/91 (87.9)
Yes	7/65 (10.8)	4/26(15.4)	11/91 (12.1)
History colon polyps			
NO	176/261 (67.4)	76/120 (63.3)	243/372 (65.3)
Yes	85/261(32.6)	44/120 (36.7)	129/372 (34.7)
Cancer status			
Tumor free	139/267 (52.1)	58/125 (46.4)	197/392 (50.3)
With tumor	128/267 (47.9)	67/125 (53.6)	195/392 (49.7)

### microRNAs selection and signature building

First, we performed differentially expressed microRNAs analysis between 441 tumor tissues and eight adjacent tissues. Then, the 441 COAD cases in TCGA database were randomly divided into a test series (N = 300) and a validation series (N = 141). Next, those differentially expressed microRNAs were analyzed using the univariate and multivariate Cox proportional hazards regression analysis in the test series. Based on the expression and coefficient of microRNAs, an optimal microRNAs prognostic signature was established and validated in both the validation and entire series. The microRNA-based risk score formula was constructed as follows:

$$ \text{Risk}\,\text{score}\,\text{ = }\,\text{ }\sum\limits_{i = 1}^{n} {Coe} \;i\,*\;\mathop {\text{EV}}\nolimits_{\text{i}} , $$

In this formula, n represents the number of microRNAs, Coei indicates the coefficient of every microRNA in the result of multivariate Cox regression analysis, and EVi represents the expression level of the every microRNA.

### mRNA and lncRNA signature building

Differentially expressed mRNA and lncRNA were selected to carry out univariate and multivariate Cox analysis. Then, we established mRNA signature and lncRNA signature based on the expression and coefficient of mRNAs and lncRNAs respectively. The risk score formula as follows:

$$ \text{Risk}\,\text{score}\,\text{ = }\,\text{ }\sum\limits_{i\, = 1}^{n} {Coe} \;i\,*\;\mathop {\text{EV}}\nolimits_{\text{i}} , $$

In this formula, n represents the number of mRNAs or lncRNAs, Coei indicates the coefficient of every mRNA or lncRNA in the result of multivariate Cox regression analysis, and EVi represents the expression level of the every mRNA or lncRNA.

### Target gene prediction and functional enrichment analysis

The target genes of these prognostic miRNAs was predicted using Targetscan (http://www.targetscan.org/) and miRDB (http://www.mirdb.org/). The overlapping target genes in these two databases were considered as miRNA-target genes and used for further analysis. Functional enrichment analysis including cellular component (CC), molecular function (MF), biological process (BP) and Kyoto Encyclopedia of Genes and Genomes (KEGG) pathway analysis were performed by STRING database (https://string-db.org/cgi/input.pl) and visualized using GraphPad Prism7 (GraphPad Software Inc., La Jolla, CA).

### Statistical analysis

Differentially expressed microRNAs, mRNA and lncRNA between the COAD patients and corresponding adjacent tissue samples were calculated by the ‘edgeR’ package. Volcano plot was calculated and depicted by “volcano” R package. Venn diagram was drew using online website (http://bioinformatics.psb.ugent.be/webtools/venn/). The OS were analyzed by Kaplan–Meier survival curve analysis and calculated by the log-rank test. The time-dependent receiver operating curve (ROC) was performed to assess the sensitivity and specificity of the signature prognosis prediction.

The Kaplan–Meier survival curve analysis of miRNA-target genes was performed using ualcan database (http://ualcan.path.uab.edu/index.html). All *P* value (*P *< 0.05) was determined statistically significant. All statistical analysis was performed with R language (https://www.r-project.org/, v3.5.1), SPSS 24.0 software (SPSS Inc., Chicago, IL) and GraphPad Prism7 (GraphPad Software Inc., La Jolla, CA).

## Results

### Screening ten microRNAs as potential prognostic markers for COAD

We identified 358 significantly differentially expressed microRNAs between 443 COAD tissues and 8 adjacent tissues (|log_2_FC| > 1, *P *< 0.05), including 223 upregulated and 135 downregulated microRNAs. Further, Univariate Cox regression analysis was performed to find out microRNAs related to patients’ prognosis in test series (N = 300). The result showed that 29 microRNAs were significantly related to the overall survival of patients with COAD (All *P *< 0.05, Table 2). To improve the precise of prognostic effect of microRNAs, those 29 candidate microRNAs were further analysis by multivariate Cox analysis in the test series. Finally, a total of 10 microRNAs were filtered as the candidate factors obviously associated with the prognosis of COAD (All *P *< 0.05, Table 3). The differentially expression level of these ten microRNAs in paired COAD patients tissue were shown Additional file 1: Figure S1a, and the microRNAs expression level in 441 COAD patients and eight adjacent normal tissues were shown in Additional file 1: Figure S1b. These results showed that six of these ten microRNAs were significantly higher expression in COAD cancer, and four of them were significantly lower expression in COAD cancer (All *P* < 0.05).

**Table 2:** Univariable Cox regression analysis to access the prognostic value of each microRNA

Gene	HR^a^	Z	*P* value^b^
hsa-miR-561	1.446513414	3.061382338	0.002203176
hsa-miR-4999	1.455114828	2.695463158	0.007029085
hsa-miR-197	1.724755289	2.687503003	0.007198846
hsa-miR-3189	0.652731227	− 2.624955746	0.008666023
hsa-miR-92a-2	0.688316473	− 2.608519806	0.009093475
hsa-miR-552	0.854514732	− 2.599786264	0.009328184
hsa-miR-92a-1	0.692360881	− 2.590209036	0.009591767
hsa-miR-3677	0.733293138	− 2.541856222	0.011026552
hsa-miR-31	1.156774428	2.537629414	0.01116061
hsa-miR-887	1.435971429	2.483541477	0.013008318
hsa-miR-126	1.611809612	2.478084312	0.013208993
hsa-miR-34b	1.305240441	2.423297739	0.015380315
hsa-miR-32	1.562669545	2.398135451	0.01647877
hsa-miR-200a	0.704267099	-2.365202179	0.01802023
hsa-miR-3199-2	1.432538638	2.318025641	0.020447928
hsa-miR-29b-2	1.43193496	2.317641614	0.020468807
hsa-miR-210	1.197112875	2.311015882	0.020831978
hsa-miR-29b-1	1.419044842	2.302408969	0.02131212
hsa-miR-6854	0.728782265	− 2.238559963	0.025184562
hsa-miR-328	1.460765612	2.213610222	0.026855604
hsa-miR-153-2	1.243537182	2.154273514	0.031218729
hsa-miR-3917	0.771090176	− 2.125123704	0.033576292
hsa-miR-576	1.38364903	2.082214899	0.037322845
hsa-miR-149	1.278012985	2.081591901	0.037379762
hsa-miR-323a	1.338950369	2.078569517	0.037656935
hsa-miR-1277	1.320851357	2.054244282	0.039952054
hsa-miR-501	0.753234657	− 2.009449019	0.044489536
hsa-miR-3605	1.285862924	1.999104141	0.045597087
hsa-miR-615	1.152363534	1.960122196	0.04998151

*HR* Hazard ratio

^a^Values  > 1.0 indicate that expression is positively associated with poor survival

^b^Likelihood ratio test *P* value

**Table 3:** The HR and *P* value of each gene

Gene	Coefficient^a^	HR^b^	*P* value^c^
hsa-miR-3199-2	0.5924	1.8082	0.00081
hsa-miR-4999	0.4152	1.5146	0.00447
hsa-miR-3917	− 0.3905	0.6767	0.00634
hsa-miR-210	0.2422	1.274	0.00771
hsa-miR-561	0.3264	1.386	0.0091
hsa-miR-6854	− 0.3851	0.6804	0.01656
hsa-miR-887	0.3766	1.4574	0.01735
hsa-miR-197	0.5059	1.6585	0.02686
hsa-miR-3189	− 0.3412	0.7109	0.04269
hsa-miR-32	0.3729	1.4519	0.04437

*HR* Hazard ratio

^a^Values  > 0.0 and ^b^Values  > 1.0 indicate that expression is positively associated with poor survival

^c^Likelihood ratio test *P* value

### Identifying a ten-microRNA signature as potential prognostic indicator of COAD

**Fig. 1:**
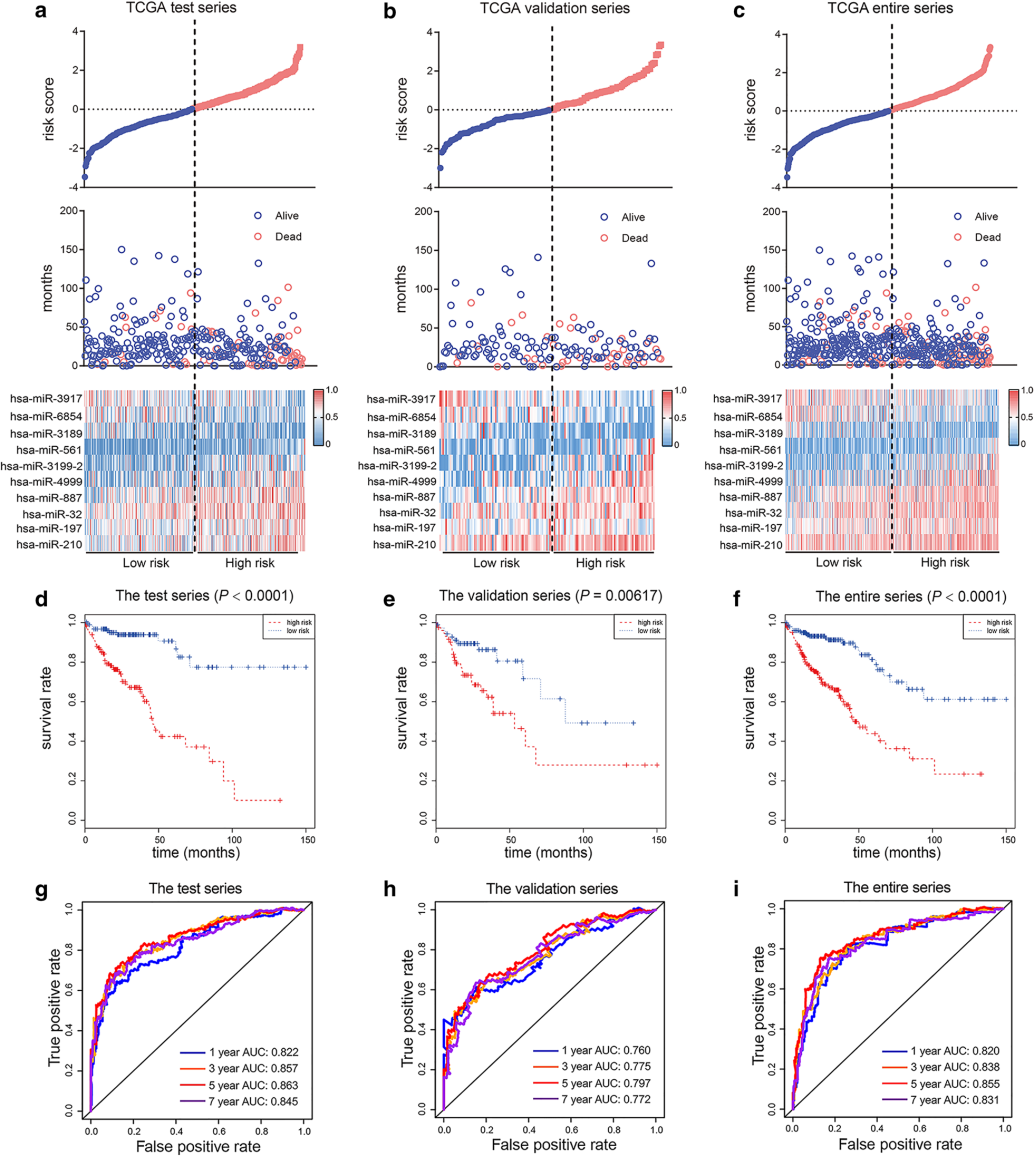
Identification of ten-microRNA signature associated with prognosis of patients. Risk score distribution, patients’ status and heatmap of ten microRNAs expression in test series (**a**), validation series (**b**) and entire series (**c**). Kaplan–Meier analysis of the ten-microRNA signature in test series (**d**), validation series (**e**) and entire series (**f**). Time-dependent ROC analysis of the sensitivity and specificity of the ten-microRNA signature in test series (**g**), validation series (**h**) and entire series (**i**)

To optimize the predictive microRNAs profile, the filtered ten microRNAs were used to construct a predictive microRNAs signature. Based on the expression levels of these ten microRNAs and their coefficient assessed by multivariate Cox analysis, a novel risk score formula was established as follows: Risk score = (0.5059*hsa-miR-197) + (0.3729*hsa-miR-32) + (− 0.3905*hsa-miR-3917) + (0.3766*hsa-miR-887) + (0.5924*hsa-miR-3199-2) + (0.4152*hsa-miR-4999) + (− 0.3412*hsa-miR-3189) + (− 0.3851*hsa-miR-6854) + (0.3264*hsa-miR-561) + (0.2422*hsa-miR-210). Among these microRNAs, the coefficients of those seven microRNAs (hsa-miR-197, hsa-miR-32, hsa-miR-887, hsa-miR-3199-2, hsa-miR-4999, hsa-miR-561 and hsa-miR-210) were positive, manifesting their COAD driving effect, while the coefficients of other three microRNAs (hsa-miR-3917, hsa-miR-3189 and hsa-miR-6854) were negative, indicating their COAD protecting effect. Based on the prognostic model formula, we calculated and ranked the risk score of each patient. The distribution of risk score, survival status of patients and the expression profiles of ten microRNAs in the test series were shown in Fig. 1a. The results showed that the patients with high risk score have shorter survival time than those with low risk score. The expression levels of seven risky microRNAs were higher in the patients with high risk scores, while the expression levels of three protective microRNAs were higher in the patients with low risk scores. And similar results were also observed in both validation (N = 141) and entire series (N = 441, Fig. 1b, c).

**Fig. 2:**
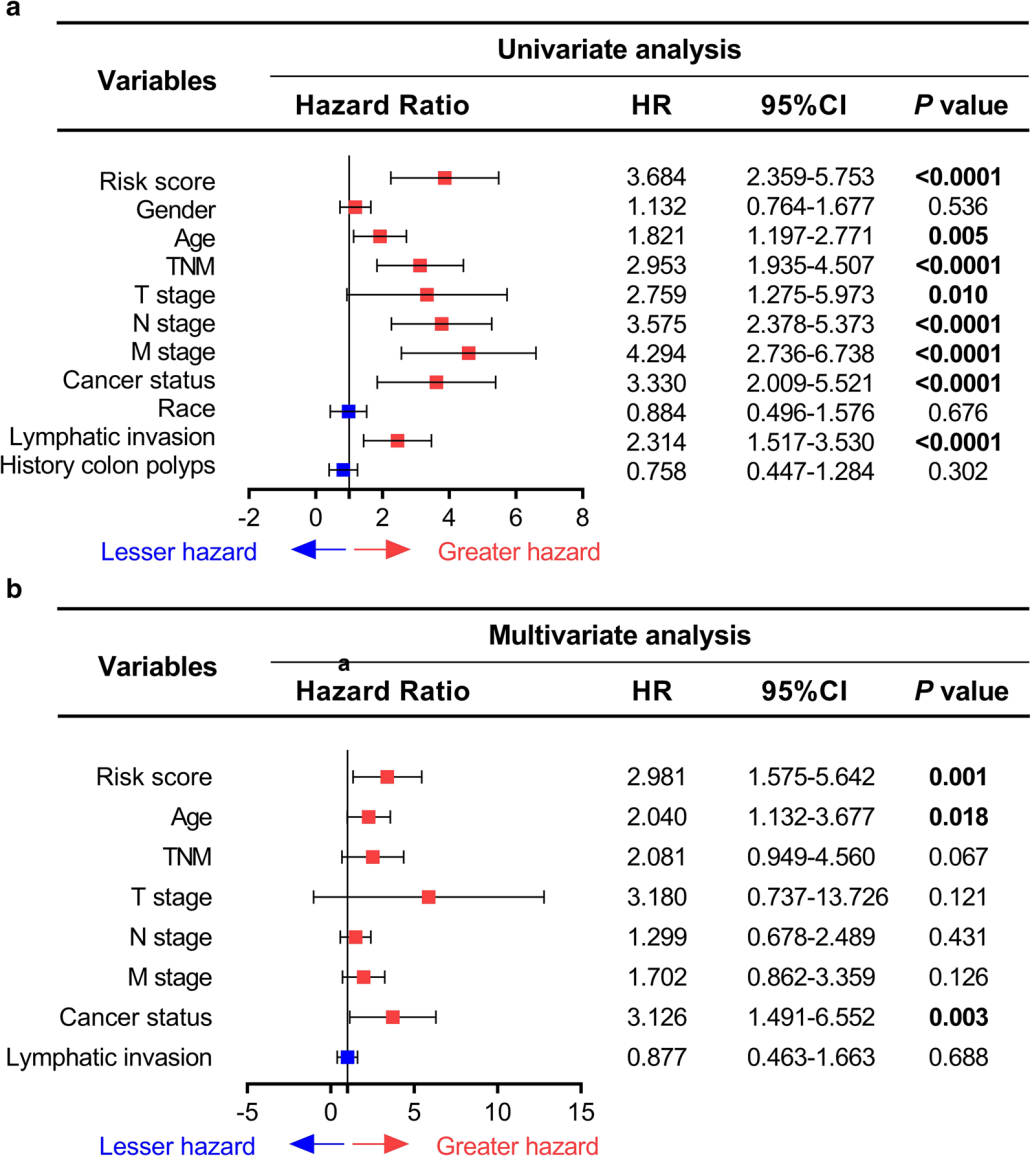
Univariate and multivariate Cox analysis of risk score and clinical characteristics. **a** Univariate Cox analysis of risk score and clinical characteristics. **b** Multivariate Cox analysis of risk score and clinical characteristics. *P* value with Bold represents *P *< 0.05. Red represents the factor was risky factor and blue represents the factor was protective factor

Then, in order to explore the prognostic value of the ten-microRNA signature, a total of 300 patients in test series were separated into high-risk score group (N = 150) and low-risk score group (N = 150). The results showed that the patients in high-risk group have an obviously shorter OS and poor prognosis than the patients in low-risk group (*P *< 0.0001, Fig. 2d). And similar results were also observed in both validation (*P *= 0.00617) and entire series (*P *< 0.0001, Fig. 1e, f). Then, the time-dependent ROC curve results from three series showed that the prognostic accuracy of the ten-microRNA signature was 0.822, 0.760 and 0.820 at 1 year; 0.857, 0.775 and 0.838 at 3 year; 0.863, 0.797 and 0.855 at 5 year; 0.845, 0.772 and 0.831 at 7 year, respectively. (Fig. 1g–i). These results suggested that the ten-microRNA signature had high specificity and sensitivity and could predict the prognosis of patients with COAD effectively. Taken together, this ten-microRNA signature is a meaningful potential biomarker to predict prognosis of COAD patients.

### The ten-microRNA signature is a prognostic indicator independent to other clinical characteristics

To further confirm the predictive effect of the ten-microRNA signature and other clinical characteristic on the prognosis, the univariate and multivariate Cox analysis were performed in entire series. The result of univariate Cox analysis showed that the ten-microRNA signature and some clinical characteristics (including age, TNM stage, T stage, N stage, M stage, Cancer status and Lymphatic invasion) were obviously correlated with the prognosis of COAD (Fig. 2a and Table 4). Furthermore, the multivariate Cox analysis showed that the ten-microRNA signature is still an independent prognostic factor (HR = 2.981, 95%CI 1.575–5.642, *P *= 0.001) (Fig. 2b and Table 4). The other conventional clinical characteristics, such as age (HR = 2.040, 95%CI 1.132–3.677, *P *= 0.018) and Cancer status (HR = 3.126, 95%CI 1.491–6.552, *P *= 0.003), were also proven to be independent factors associated with the overall survival (Fig. 2b and Table 4). To further verify prognostic ability of clinical characteristics, we performed Kaplan–Meier analysis. The results were consistent with univariate Cox analysis results and showed that age (*P *= 0.0116), TNM stage (*P *< 0.0001), T stage (*P *= 0.0032), N stage (*P *< 0.0001), M stage (*P *< 0.0001), cancer status (*P *< 0.0001) and Lymphatic invasion (*P *= 0.0004) were associated with prognosis of COAD patients (Additional file 2: Figure S2a–i).

**Table 4:** Univariable and multivariable Cox analysis of the risk score and clinical information in entire series

Variables	Univariate analysis		Multivariate analysis	
	HR^a^ (95%CI)	*P* value^b^	HR^a^ (95%CI)	*P* value^b^
Risk score				
Low	1 (Reference)		1 (Reference)	
High	3.684 (2.359–5.753)	*< 0.0001*	2.981 (1.575–5.642)	*0.001*
Gender				
Female	1 (Reference)			
Male	1.132 (0.764–1.677)	0.536		
Age				
< 68	1 (Reference)		1 (Reference)	
≥ 68	1.821 (1.197–2.771)	*0.005*	2.040 (1.132–3.677)	*0.018*
TNM				
I + II	1 (Reference)		1 (Reference)	
III + IV	2.953 (1.935–4.507)	*< 0.0001*	2.081 (0.949–4.560)	0.067
T stage				
T0 + 1	1 (Reference)		1 (Reference)	
T2 + 3	2.759 (1.275–5.973)	*0.010*	3.180 (0.737–13.726)	0.121
N stage				
N0 + 1	1 (Reference)		1 (Reference)	
N2	3.575 (2.378–5.373)	*< 0.0001*	1.299(0.678–2.489)	0.431
M stage				
M0	1 (Reference)		1 (Reference)	
M1	4.294(2.736–6.738)	*< 0.0001*	1.702(0.862–3.359)	0.126
Cancer status				
No	1 (Reference)		1 (Reference)	
Yes	3.330 (2.009–5.521)	*< 0.0001*	3.126 (1.491–6.552)	*0.003*
Race				
Non-white	1 (Reference)			
White	0.884 (0.496–1.576)	0.676		
Lymphatic invasion				
No	1 (Reference)		1 (Reference)	
Yes	2.314(1.517–3.530)	*< 0.0001*	0.877 (0.463–1.663)	0.688
Microsatallite instability				
No	1 (Reference)			
Yes	26.379 (0.127–5479.857)	0.229		
History colon polyps				
No	1 (Reference)			
Yes	0.758 (0.447–1.284)	0.302		

Italic, significant values  < 0.05

*HR* hazard ratio; 95%CI, 95% confidence interval

^a^Values  > 1.0 indicate that expression is positively associated with poor survival

^b^Likelihood ratio test *P* value

**Fig. 3:**
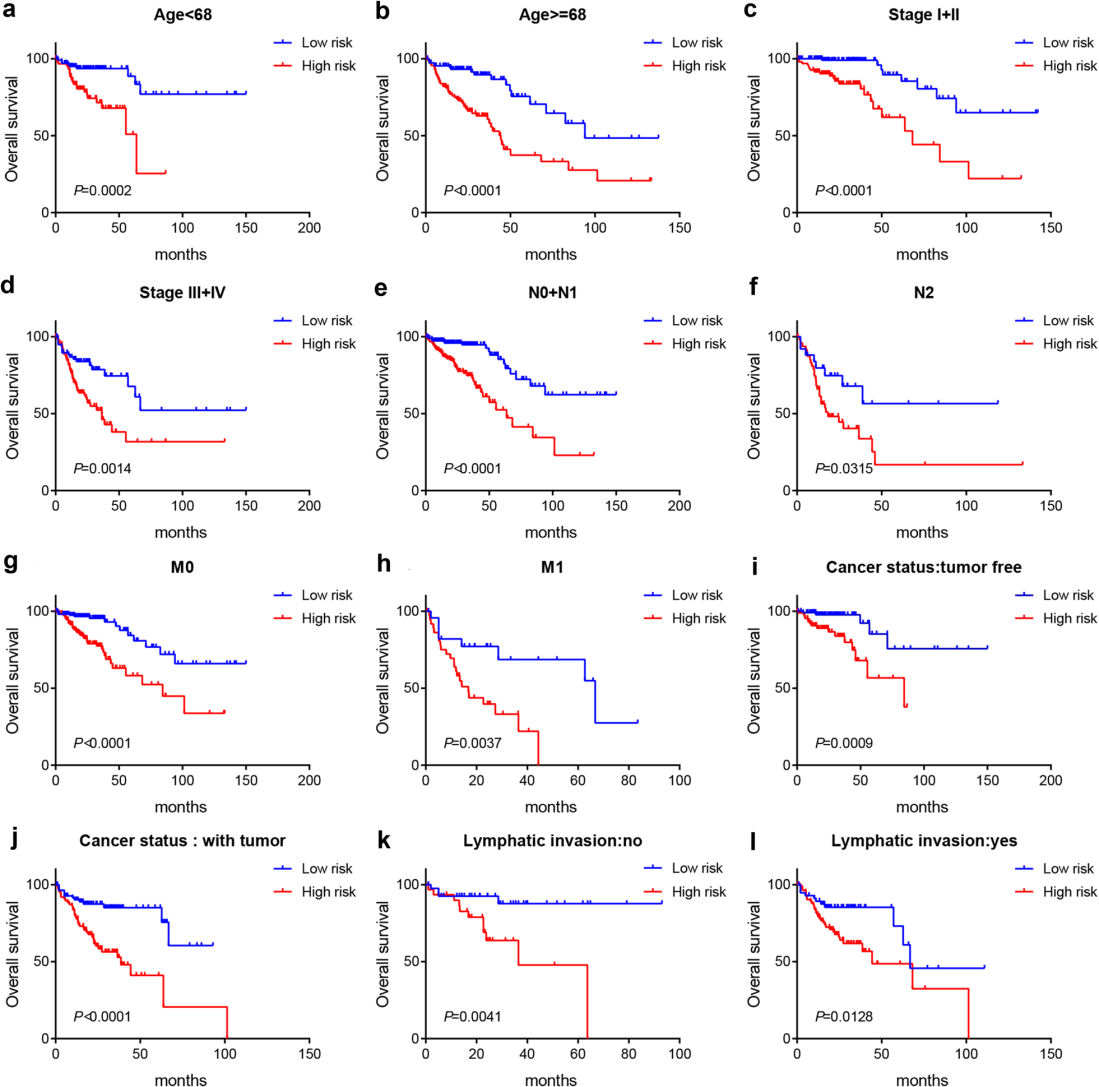
Stratification analysis of the ten-microRNA signature. Kaplan–Meier analysis of the ten-microRNA signature for patients with different clinical characteristics, including age < 68 (**a**) or age ≥ 68 (**b**), stage I + II (**c**) or stage III + IV (**d**), N 0 + 1 stage (**e**) or N2 stage (**f**), M0 stage (**g**) or M1 stage (**h**), tumor free (**i**) or with tumor (**j**) and no lymphatic invasion (**k**) or lymphatic invasion (**l**)

Furthermore, stratified analysis were performed to further evaluate whether the ten-microRNA signature exhibit predictive effect within same clinical characteristics. We stratified patients into different group based on age (< 68 or > = 68), TNM stage (I + II or III + IV), N stage (N0 + N1 or N2), M stage (M0 or M1), Cancer status (tumor free or with tumor) and Lymphatic invasion (yes or no). These results showed that the ten-microRNA signature can still separate patients into high-risk or low-risk group, and patients in high-risk group have shorter OS and poor prognosis than those in low-risk group (Fig. 3a–l). Taken together, these results demonstrated that this ten-microRNA signature was independent risk factors for survival prediction of COAD patients and could stratify patients from different group into subtypes with different prognosis.

### The ten-microRNA signature perform better in survival prediction than other signature

**Fig. 4:**
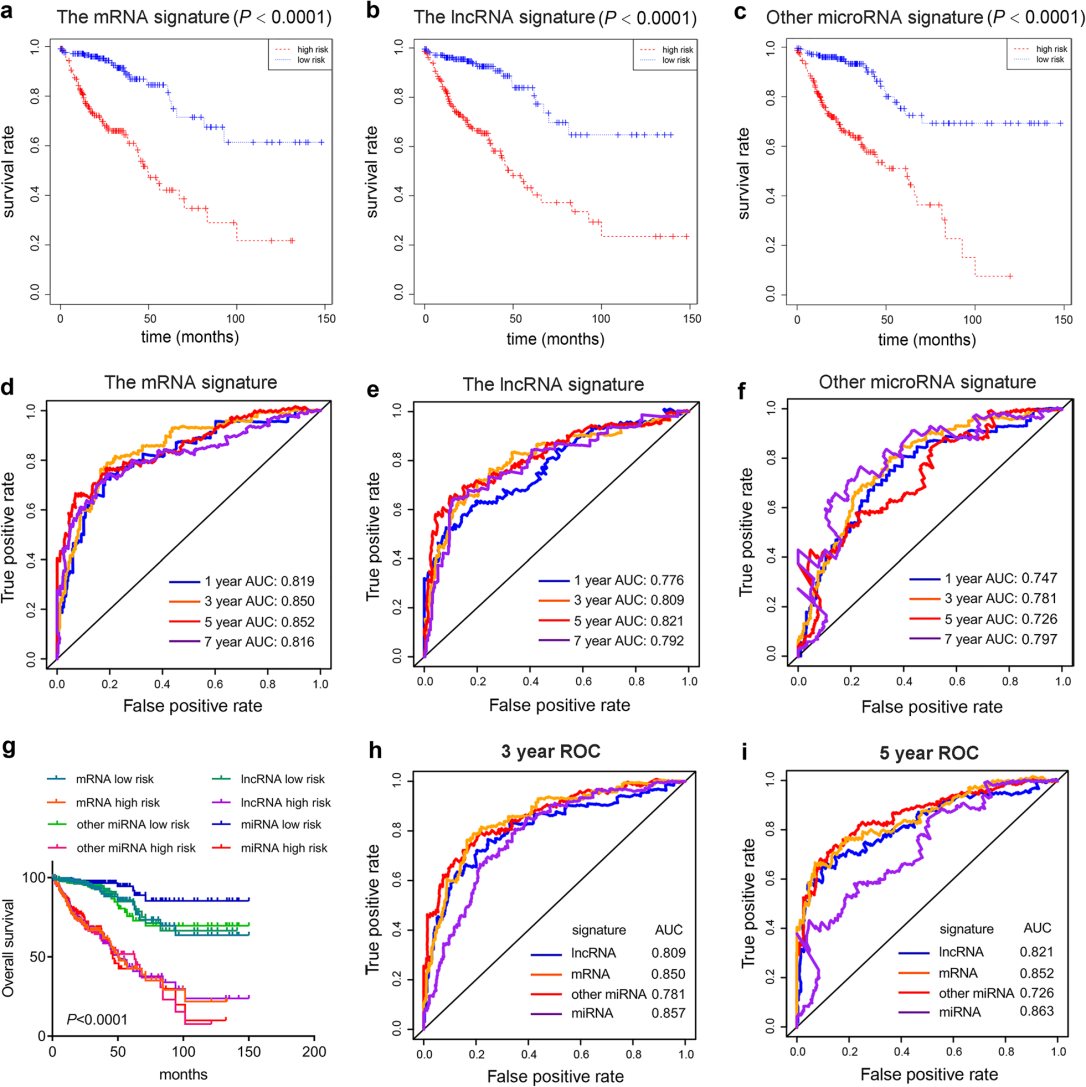
Comparison of Kaplan–Meier and time-dependent ROC analysis of our microRNA signature with other signature. Kaplan–Meier analysis of the mRNA signature (**a**), lncRNA signature (**b**) and other microRNA signature (**c**). Time-dependent ROC analysis of the sensitivity and specificity of the mRNA signature (**d**), lncRNA signature (**e**) and other microRNA signature (**f**). **g** Comparison of Kaplan–Meier analysis of our microRNA signature, mRNA signature, lncRNA signature and other microRNA signature. **h** Comparison of 3-year ROC analysis of the sensitivity and specificity of our microRNA signature, mRNA signature, lncRNA signature and other microRNA signature. **i** Comparison of 5-year ROC analysis of the sensitivity and specificity of our microRNA signature, mRNA signature, lncRNA signature and other microRNA signature

To compare the predictive effect between miRNA signature and other signature, we first performed differentially mRNA and lncRNA expression analysis between patients and adjacent tissues. Then, those different expression mRNA and lncRNA were subjected to univariate and multivariate analysis. Furthermore, we established a mRNA signature and lncRNA signature as follows: mRNA risk score signature = (− 0.2979*CPT2) + (− 0.1522*PPARGC1A) + (0.2133*UCN) + (0.0934*OFCC1) + (0.1621*ATP6V1B1) + (0.1110*LRP2) + (0.1052*MAGEA1) + (0.0677*HOXC6) + (− 2.0747*RNU11) + (− 0.2704*CD1B) + (0.2944*TYRO3P) and lncRNA risk score signature = (0.07464* RP11-400N13.2) + (0.12306* RP11-742B18.1) + (0.23462* RP11-54O7.17) + (0.40607* AC002076.10) + (0.29414* CTC-573N18.1) + (0.22067* RP11-626H12.2) + (0.31037* RP11-122K13.7) + (0.19574* RP11-114H21.2). Then, we performed Kaplan–Meier curve analysis (Fig. 4a, b) and time-dependent ROC curve with mRNA signature and lncRNA signature (Fig. 4d, e). These results showed both the mRNA signature and lncRNA signature exhibited their prognostic value as biomarker for COAD and have high specificity and sensitivity for OS of COAD. In addition, we performed KM plot curve and time-dependent ROC curve with other microRNA signature in previous study (Fig. 4c, f). Next, we compare predictive performance between our microRNA signature and other signature. The Kaplan–Meier curve analysis result showed that patients with our low risk score have significant better survival than those with other low risk score. The time-dependent ROC analysis results showed that our microRNA signature have obvious higher AUC than other signature in 3 year and 5 year OS. Taken together, our ten-microRNA signature is a superior indicator for prognosis of COAD patients.

### High risk score associates with advanced TNM stage, lymphatic metastasis and invasion of COAD patients

**Fig. 5:**
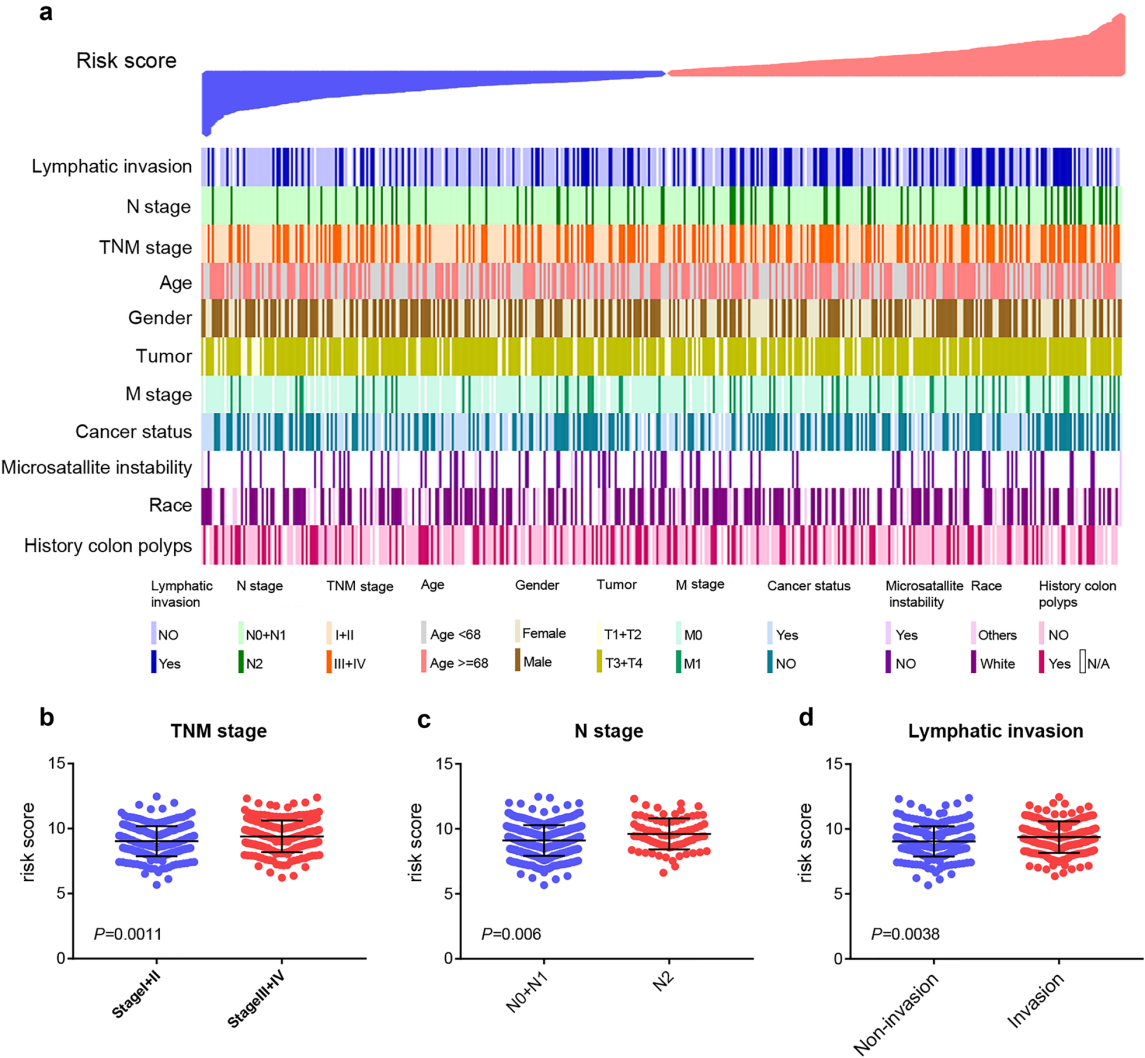
Relationship between clinical characteristics and the risk score. **a** The clinical characteristics of patients arranged by the increasing risk score. The distribution of risk score in patients stratified by TNM stage (**b**), N stage (**c**) and lymphatic invasion (**d**)

We further investigated if there was a relationship between the ten-microRNA signature and clinical pathological characteristics. The result showed that TNM stage (*P *= 0.009), N stage (*P *= 0.003) and Lymphatic invasion (*P *= 0.001) were significantly related to the ten-microRNA signature and patients with high risk score were mainly enriched in stage III + IV, N2 stage and Lymphatic invasion (Table 5). To visualize the relationship between the ten-microRNA signature and other clinical characteristic, we ranked patients according to their risk score, and found obviously asymmetric distribution of the TNM stage, N stage and Lymphatic invasion (Fig. 5). The result showed that these clinical characteristics, advanced TNM stage (III + IV stage), higher N stage (N2 stage) and Lymphatic invasion were mainly enriched in the higher risk section (Fig. 5a). We further compared the risk score of patients separated by these clinical characteristics. These results showed that the risk score signature was higher in TNM stage III and IV, compared with TNM stage I and II (Fig. 5b). Contrast to the lower N stage, the risk score signature was higher in N2 stage (Fig. 5c). Further, the risk score signature was mainly higher in Lymphatic invasion (Fig. 5d). However, the other clinical characteristic showed no significant correlation with the ten-microRNA signature (Fig. 5a and Table 5). Taken together, these results indicated that high risk score could predict advanced TNM stage, lymphatic metastasis and invasion of COAD patients.

**Table 5:** Correlation between risk score and clinicopathological features of COAD patients

Characteristics	N	Risk score level		
		Low	High	*P* value^a^
Gender				0.063
Male	231	125	106	
Female	210	95	115	
Age (years)				0.318
≥68	237	113	124	
<68	204	107	97	
TNM stage				*0.009*
I and II	241	135	106	
III and IV	189	82	107	
T stage				0.476
T1 + T2	85	45	40	
T3 + T4	355	174	181	
N stage				*0.003*
N0 + 1	361	192	169	
N2	80	29	51	
M stage				0.054
M0	320	165	155	
M1	65	25	40	
Cancer status				0.364
Tumor free	197	104	93	
With tumor	195	94	101	
Race				0.917
Non-white	71	35	36	
White	212	103	109	
Lymphatic invasion				*0.001*
No	243	137	106	
Yes	156	62	94	
Microsatallite instability				0.206
No	80	42	38	
Yes	11	8	3	
History colon polyps				0.899
No	243	126	117	
Yes	129	66	63	

Italic, significant values < 0.05

^a^*P* values were calculated by X^2^ test

### Incorporation of risk score into N stage and lymphatic invasion better predicts prognosis of COAD patients

**Fig. 6:**
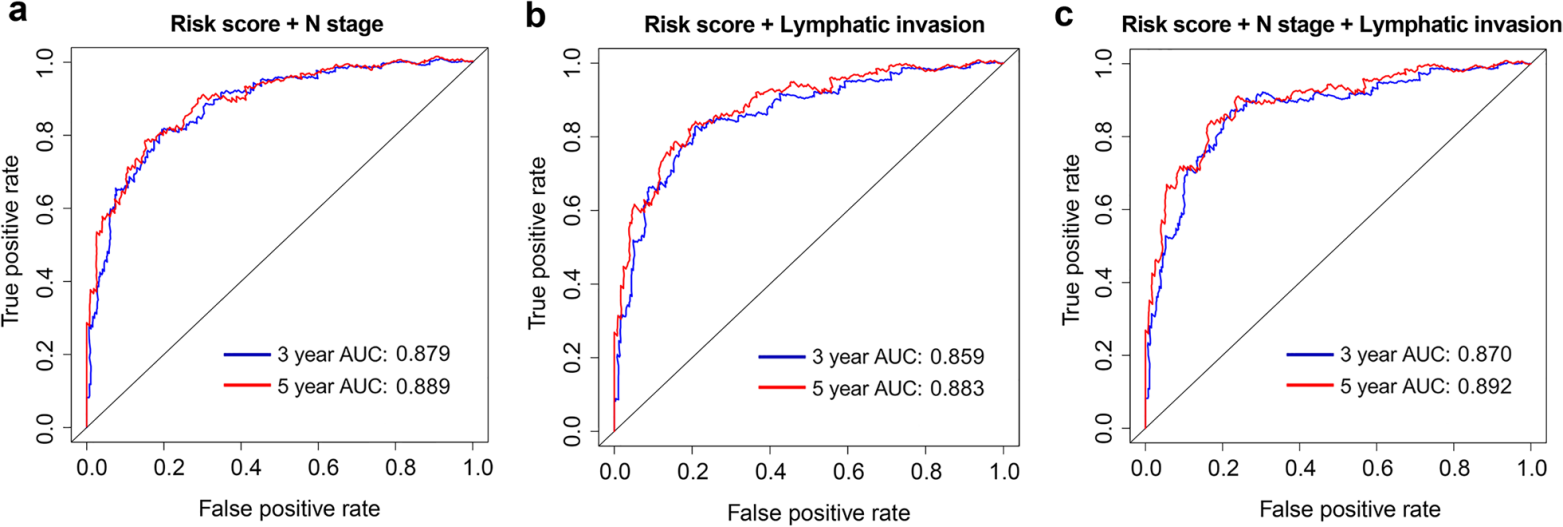
Time-dependent ROC analysis of combined the risk score and clinical characteristics. **a** Time-dependent ROC analysis of the sensitivity and specificity of combined the risk score and N stage. **b** Time-dependent ROC analysis of the sensitivity and specificity of combined the risk score and lymphatic invasion. **c** Time-dependent ROC analysis of the sensitivity and specificity of combined the risk score, N stage and lymphatic invasion

Since many studies have shown that combined biomarkers are able to improve the prognostic accuracy than a single marker. And our risk score were positively associated with N stage and lymphatic invasion. Hence, we further investigated whether the incorporation of risk score into N stage and lymphatic invasion could better predict the prognosis for OS of COAD patients. The time-dependent ROC curve analysis results showed that AUC was 0.879, 0.859 and 0.870 at 3 year; 0.889, 0.883 and 0.892 at 5 year for OS with combined risk score and N stage, combined risk score and lymphatic invasion and combined risk score, N stage and lymphatic invasion (Fig. 6). These results demonstrated that combining risk score and N stage and lymphatic invasion could better predict prognosis of COAD patients.

### Identifying miRNA-target genes associated with lymphatic metastasis and invasion and poor prognosis of COAD patients

**Fig. 7:**
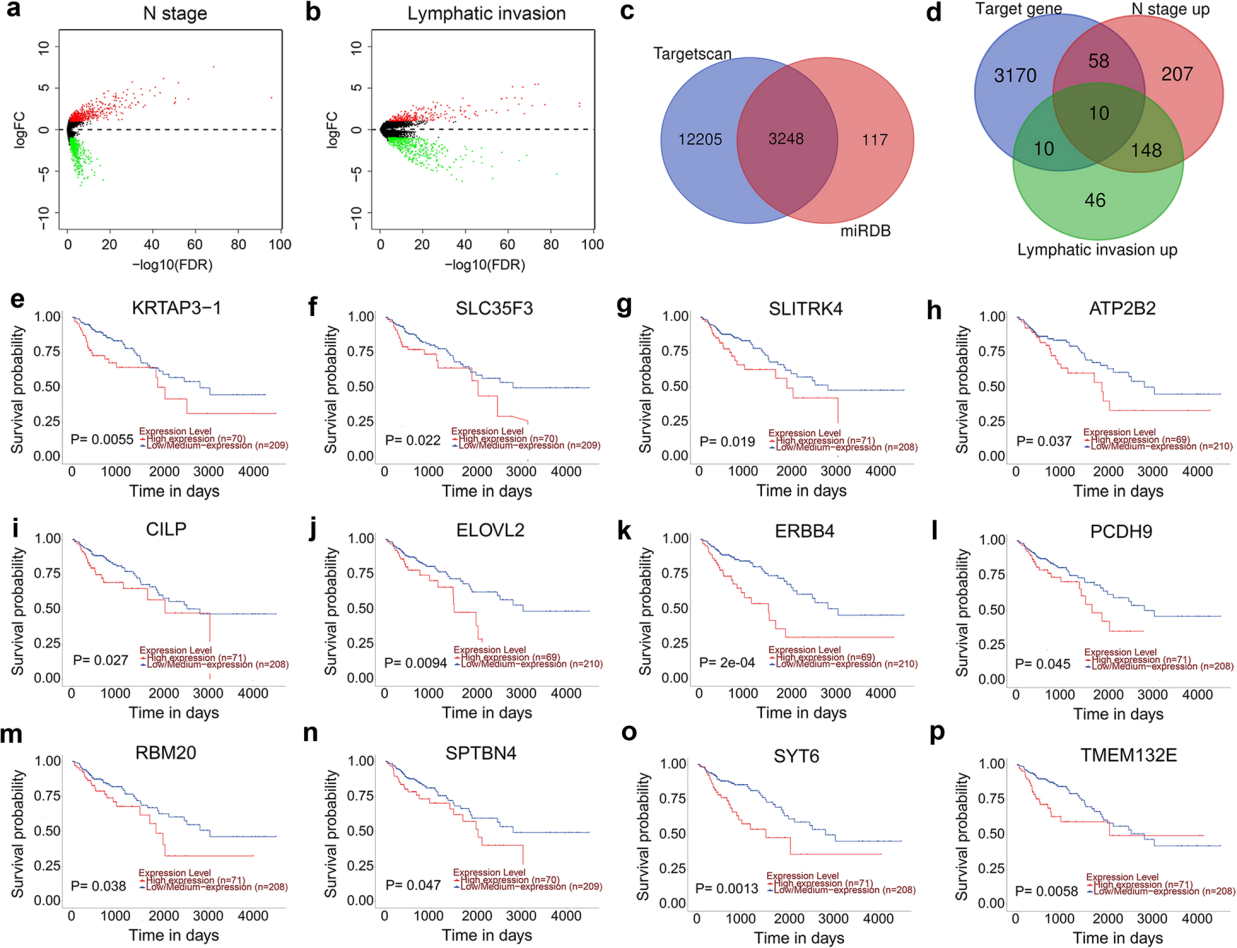
Kaplan–Meier analysis of miRNA-target genes associated with N stage and lymphatic invasion of patients. **a** Volcano plot of differentially expressed genes in COAD patients with N0 + N1 stage vs N2 stage. **b** Volcano plot of differentially expressed genes in COAD patients with non-lymphatic invasion vs lymphatic invasion. **c** The intersection of miRNA target genes predicted using Targetscan database and miRDB database. **d** The intersection of miRNA target genes, upregulated in N stage and upregulated in lymphatic invasion. **e**–**g** Kaplan–Meier analysis of miRNA-target genes upregulated both in N stage and lymphatic invasion. **h**–**p** Kaplan-Meier analysis of miRNA-target genes upregulated in N stage

To explore genes that affect lymphatic metastasis and invasion of COAD patients, we first performed differentially mRNA expression analysis between N0 + N1 stage and N2 stage, non-lymphatic invasion and lymphatic invasion respectively (Fig. 7a, b). In addition, we also investigated the target genes of the ten microRNAs using two independent miRNA target gene prediction websites: Targetscan and miRDB. Target genes overlapping in the two websites were considered as miRNA-target genes and there are 3248 target genes (Fig. 7c). Then, Venn diagram showed that in these target genes, there are 58 mRNAs were upregulated in N stage, 10 mRNAs were upregulated in lymphatic invasion and 10 mRNAs were upregulated in both N stage and lymphatic invasion. Furthermore, the Kaplan–Meier curve was performed to analyze the prognostic value of these miRNA-target genes using ualcan website. The results showed that there are 12 mRNAs were associated with prognosis of COAD patients, where *KRTAP3*-*1*, *SLC35F3* and *SLITRK4* were upregulated in both N stage and lymphatic invasion, while *ATP2B2*, *CILP*, *ELOVL2*, *ERBB4*, *PCDH9*, *RBM20*, *SPTBN4*, *SYT6* and *TMEM132E* were upregulated in N stage. Taken together, these miRNA-target genes might affect the prognosis of COAD patients through promote lymphatic metastasis and invasion of patients.

### Identifying biological pathways that the ten-microRNA signature promote lymphatic metastasis and invasion of COAD patients

**Fig. 8:**
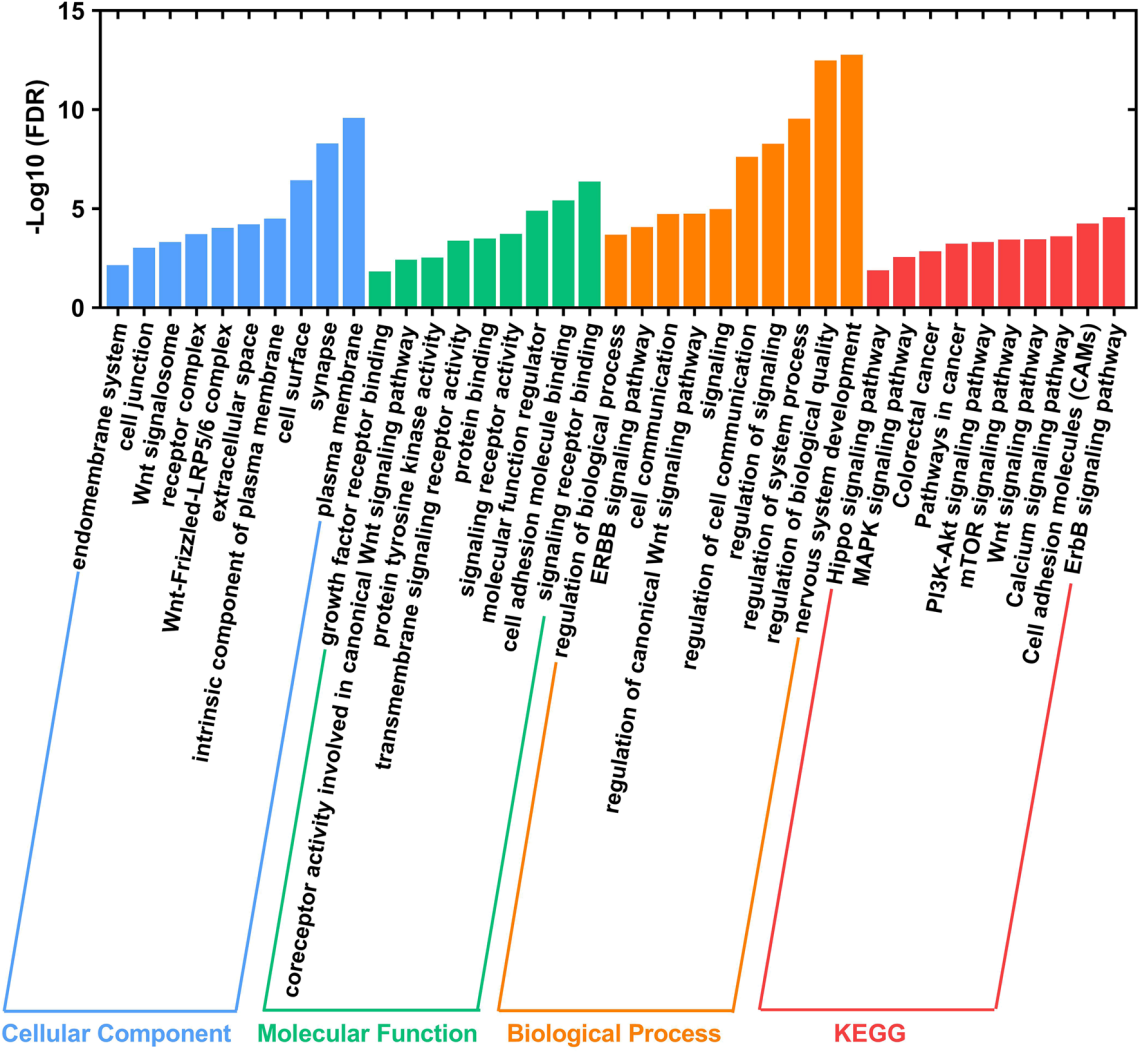
Functional enrichment analysis of miRNA-target genes upregulated in N stage or lymphatic invasion. Functional enrichment analysis including cellular component, molecular function, biological process and KEGG analysis of 78 miRNA-target genes upregulated in N stage or lymphatic invasion

We put 78 miRNA-target genes upregulated in N stage or lymphatic invasion into STRING to explore potentially biological signaling pathways correlated with the ten-microRNA signature. As shown in Fig. 8, the GO analysis results showed that these target genes were significantly enriched in the Wnt signaling pathway. The KEGG analysis result showed that these target genes were significantly associated with Hippo signaling pathway, MAPK signaling pathway, PI3K-Akt signaling pathway, mTOR signaling pathway, Wnt signaling pathway and so on. Hence, we speculated that these target genes promote progression of COAD patients mainly through Wnt signaling pathway.

## Discussion

COAD is part of colorectal cancer which is a highly heterogeneity of disease with molecular complexity [10]. This feature makes traditional clinical predictive indicators such as stage and so on not enough to predict survival for patients. Although plenty of prognosis biomarkers of COAD has been reported [2, 11], most of them are lacked accuracy and have not been validated in clinical practice. Therefore, novel biomarkers are urgently needed to provide more accurate survival prediction for COAD patients.

During the past years, microRNAs are regarded as novel disease biomarkers because of the universality and stability in cancer [12]. Therefore, in this study, we identified a ten-microRNA signature to better predict the prognosis of COAD patients. Among those selected microRNAs, seven of them (hsa-miR-32, hsa-miR-197, hsa-miR-210, hsa-miR-3189, hsa-miR-3917, hsa-miR-4999, and hsa-miR-6854) were previous reported to have critical roles in colorectal carcinoma [13–16]. Three of them (hsa-miR-3189, hsa-miR-3917 and hsa-miR-6854) appear to be protective factors for colon cancer consistent with previous study and four of them (hsa-miR-32, hsa-miR-197, hsa-miR-210 and hsa-miR-4999) display to be risky factors for colon cancer also consistent with previous study. The consistency with the reported microRNAs indicated that the selected ways in our study were reasonable. The left three selected microRNAs (hsa-miR-887, hsa-miR-3199-2 and hsa-miR-561) have not been identified to be correlated with the prognosis of colon cancer, but these microRNAs have been reported to be correlated with the development, progression, and prognosis of other cancers. For instance, has-miR-887 was reported to be associated with the metastasis and progression of prostate cancer [17]. has-miR-3199-2 was found to be a prognostic biomarker for papillary renal cell carcinoma [18], and hsa-miR-561 was reported to inhibit gastric cancer cell proliferation and invasion by downregulating c-Myc expression [19]. These results indicated that these microRNAs might also play an important role in colon cancer. In addition, among our screened microRNAs, miR-32, miR-4999, miR-3189, miR-6854, miR-561 and miR-210 were significantly upregulated in COAD patients (Additional File 1: Figure S1a, b), suggesting these microRNAs might exhibit diagnostic value in COAD.

By applying this microRNA signature to the patients, a significantly risk stratification for patients’ outcome was observed between survival curves of patients with high-risk or low-risk scores. Patients in the high-risk group have a significantly shorter survival and poor prognosis than those in the low-risk group. These results suggested that prognostic value of the microRNA signature is robust and reliable for survival prediction in ccRCC patients. Besides, univariate and multivariate Cox analysis results demonstrated that our microRNA signature is an independent risk factor for prognosis of COAD patients.

At present, although several biomarkers including secretory proteins, mRNA and lncRNA signatures have been used for diagnosis and prognosis in a variety of cancers, they still have themselves limits respectively [20–22]. Generally, compared with protein biomarkers, RNA biomarkers are more sensitivity and specificity, whose cost are lower. These results provide a good idea for us to research early diagnosis and prognosis monitoring of patients from the perspective of RNA. Regrettably, mRNA and lncRNA is easily degraded during extraction. However, miRNA as an alternative biomarker have a promising clinical value in diagnosis and prognosis monitoring of patients. More importantly, our microRNA signature have higher accuracy for OS than mRNA, linRNA and other miRNA signature. Therefore, our study provide a novel biomarker with superiority to predict the prognosis of COAD patients.

Tumor metastasis is the chief cause of death in the vast majority of cancer patients including COAD and is indicative for poor prognosis [23, 24]. However, it is strongly correlated with initial tissue invasion at the primary tumor site [25]. Therefore, it is need to point out that novel therapeutic strategies still need to explore to avoid metastasis. In our study, high risk score indicated advanced TNM stage, higher N stage and lymphatic invasion, suggesting our signature could affect the progression of COAD patients. Furthermore, we identified some miRNA-target genes that promote lymphatic metastasis and invasion and associate with survival of patients. These target genes might exhibit a helpful indicator for lymphatic metastasis and invasion and prognosis of COAD patients. Among these target genes, *ERBB4* has been reported to be associated with colon cancer metastasis, which was consistent with our results [26]. The left of target genes might be novel potential therapeutic targets for patients with lympghatic metastasis and invasion.

Wnt family genes play essential roles in human tumorigenesis. The Wnt signaling pathway could involve in cell proliferation, migration and fate during embryonic development. In adulthood, Wnt signaling pathway exhibit a critical role in regulating homeostasis and self-renewal of tissues. Particularly, in the intestinal epithelium, Wnt signaling pathway promotes proliferation and/or differentiation of stem cells in the intestinal crypts. This is why Wnt signaling pathway play an important role in colon carcinoma [27]. In our study, the GO and KEGG analysis were mainly enriched in Wnt signaling pathway. Hence, we believed that these miRNA-target genes might affect the progression of COAD patients through Wnt signaling pathway.

In addition, a growing number of studies suggest that combined molecular biomarkers with clinical characteristics are able to improve the prognostic accuracy for patients than a single biomarker. Therefore, we incorporated our signature with N stage and lymphatic invasion, which resulted in improved predictive accuracy in OS of COAD patients compared to using signature alone. Thus, our microRNA signature could serve as a help indicator to predict prognosis of patients, especially when combined with N stage and lymphatic invasion.

However, there are also some limitations in this study. First, the prognostic signature of microRNA-based expression were identified by reasonable statistical approaches, but the results was only verified in TCGA database and not verified in clinical practice. Then, although we have some clinical characteristics information in this analysis, several important factors including alcohol consumption, food style, smoking history, and treatment information (surgery, chemotherapy, and radiotherapy) were not available in TCGA database, and we could not control those factors that might cause biases in our analysis. Final, The large scale studies are needed to exam our signature before the ten-microRNA signature can be applied in the clinical practice.

## Conclusions

In this study, we established a ten-microRNA signature as a novel superior and independent indicator to accurately predict prognosis of COAD patients. Furthermore, we identified some miRNA-target genes that affect lymphatic metastasis and invasion and prognosis of COAD patients. These miRNA-target genes were able to be novel therapeutic therapy for patients with lymphatic metastasis and invasion. Finally, we found Wnt signaling pathway might be the significant mechanism in affecting the progression of COAD.

## Supplementary information

**Additional file 1: Figure S1.** The differentially expression of ten microRNAs. **a** The differentially expression of ten microRNAs in TCGA database between COAD tissues (N = 8) and paired adjacent tissues (N = 8). **b** The differentially expression of ten microRNAs in TCGA database between COAD tissues (N = 441) and adjacent tissues (N = 8).

**Additional file 2: Figure S2.** Kaplan–Meier analysis of clinical characteristics. Kaplan–Meier analysis of age **a**, gender **b**, TNM stage **c**, T stage **d**, N stage **e**, M stage **f**, cancer status **g**, lymphatic invasion **h**, microsatellite instability **i**.

## Data Availability

All data generated or analyzed during this study are included in this published article.

## Abbreviations

COAD: colon adenocarcinoma
TCGA: The Cancer Genome Atlas
ROC: receiver operating curve
AUC: area under the curve
OS: overall survival
