# Supplementary figures and images for: Identifying a ten-microRNA signature as a superior prognosis biomarker in colon adenocarcinoma
Source: Cancer Cell Int. 2019 Dec 30;19:360. doi: 10.1186/s12935-019-1074-9 (PMC6937800; doi:10.1186/s12935-019-1074-9)

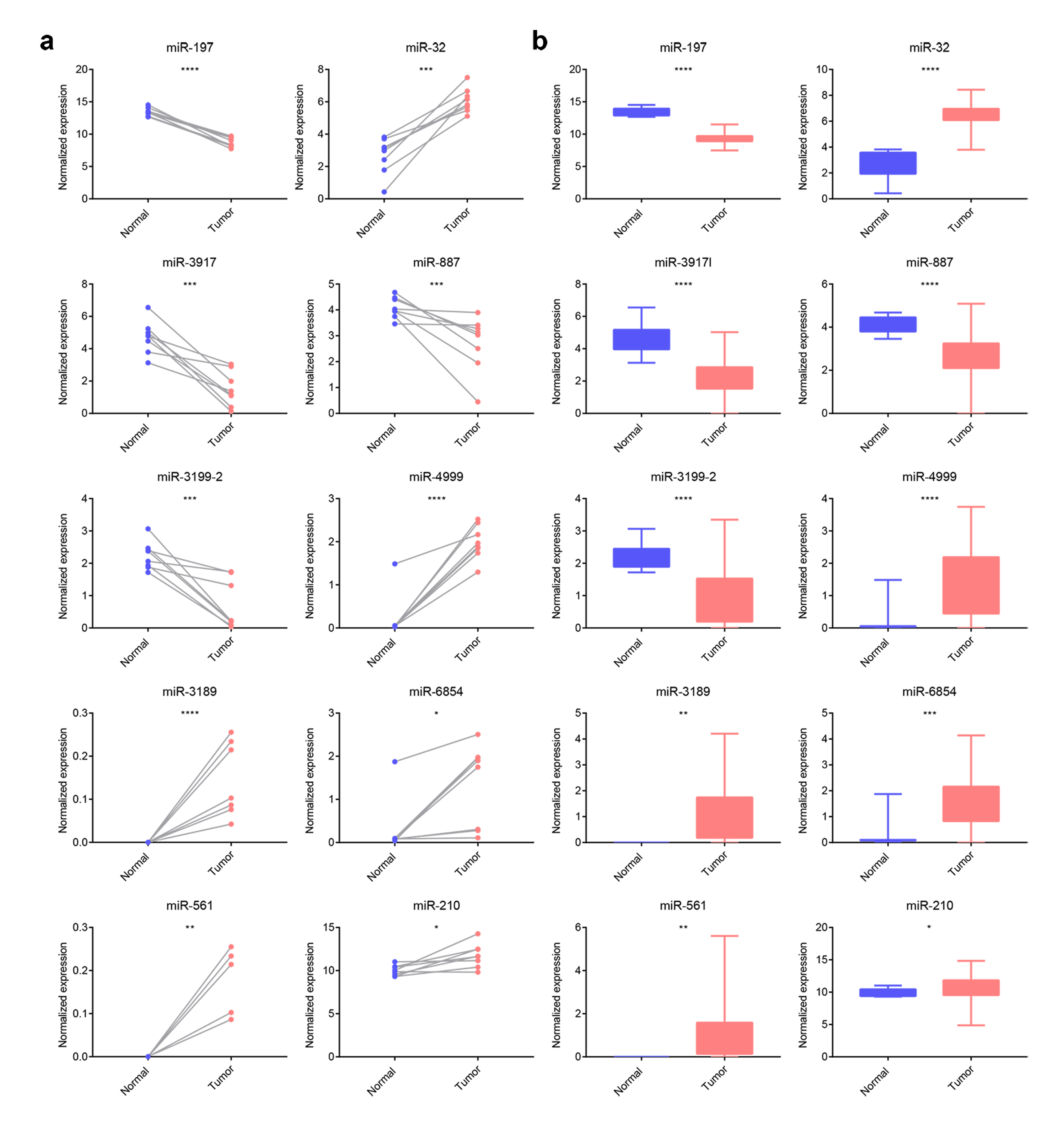

Supplement: Supplementary file 1 — Additional file 1: Figure S1. The differentially expression of ten microRNAs. a The differentially expression of ten microRNAs in TCGA database between COAD tissues (N = 8) and paired adjacent tissues (N = 8). b The differentially expression of ten microRNAs in TCGA database between COAD tissues (N = 441) and adjacent tissues (N = 8). [file 12935_2019_1074_MOESM1_ESM.tif]

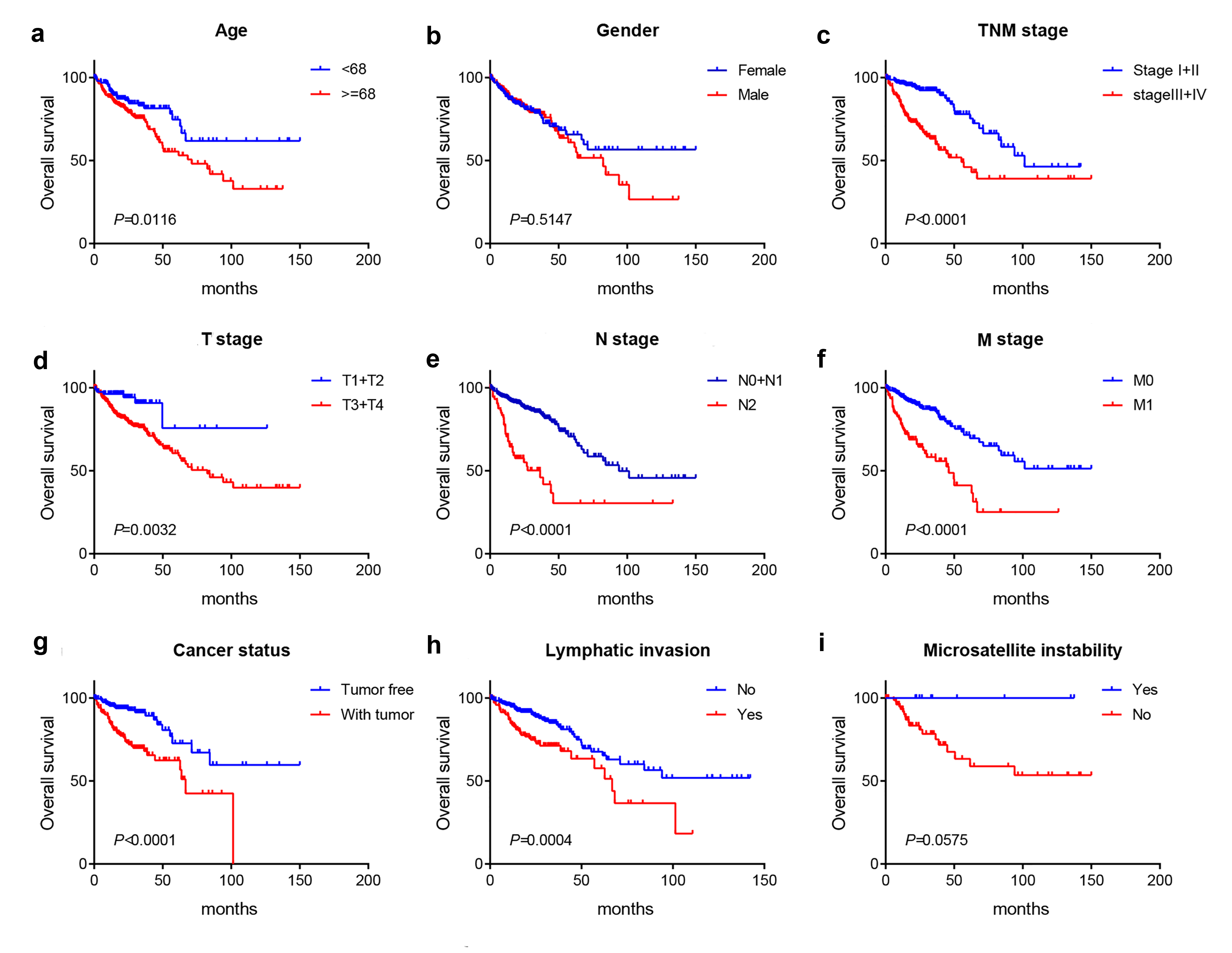

Supplement: Supplementary file 2 — Additional file 2: Figure S2. Kaplan–Meier analysis of clinical characteristics. Kaplan–Meier analysis of age a, gender b, TNM stage c, T stage d, N stage e, M stage f, cancer status g, lymphatic invasion h, microsatellite instability i. [file 12935_2019_1074_MOESM2_ESM.tif]
